# Supplementary material for: Incremental Validity of Trait Impulsivity, Dysfunctional Emotional Regulation, and Affect Lability in the Predictions of Attention Deficit Hyperactivity Disorder and Oppositional Defiant Disorder Symptoms in Adults
Source: Behav Sci (Basel). 2024 Jul 14;14(7):598. doi: 10.3390/bs14070598 (PMC11273999; doi:10.3390/bs14070598)
Supplement: Supplementary file 1 [file behavsci-14-00598-s001.zip › behavsci-3013045-supplementary.pdf]

Supplementary Table S1

Background Information of Participants

	Frequency	Percentage (if applicable)
All	525	100%
Gender		
Men	142	27.0%
Women	385	73.0%
Age (Mean; SD)		
All	32.91 (12.94)	
Men	33.74 (12.91)	t (523) = 0.898, p = .370
Women	32.60 (11.95)	
Background		
Relationship		
Single	153	29.1
Married	188	35.8
De-facto	89	17.0
Separate	10	1.9
Divorced	12	2.3
Widowed	1	.2
In relation but leaving apart	72	13.7
Education		
Primary	2	.4
Secondary/High School	149	28.4

TAFE/Trade Certificate	90	17.1
Undergraduate	170	32.4
Postgraduate	114	21.7
Employment		
Student	170	32.4
Unemployed	14	2.7
Retired	3	.6
Full Time	189	36.0
Casual / Part Time	149	28.4
Meeting ADHD symptoms threshold number		
ADHD predominantly inattention type	25	4.76
ADHD predominantly hyperactive/impulsive type	11	2.1
ADHD combined type	25	4.76
Total	61	11.62

Note. Symptom counts were based on recoding ADHD items (in the CSS) rated 0 and 1 as symptom absent, and 2 and 3 as symptom present. The threshold number was 5 for more symptoms in the respective ADHD symptom groups.

Supplementary Table S2

Descriptives, and Correlations of Study Variables

	1	2	3	4	5	6	7	8
Age (1)	1	-.04	-.17**	-.27**	-.24**	-.17**	-.10*	-.22**
Gender (2)		1	-.04	.07	.01	-.08	-.04	-.04
Trait impulsivity (3)			1	.51**	.52**	.59**	.49**	.50**
Dysfunctional emotional regulation (4)				1	.72**	.59**	.50**	.55**
Affect lability (5)					1	.63**	.57**	.63**
Inattention (6)						1	.75**	.55**
Hyperactivity/impulsivity (7)							1	.51**
Oppositional Defiant Disorder (8)							.	1
Mean	32.91	-	44.12	64.48	31.10	5.87	6.12	3.81
Standard deviation	12.94	-	8.29	22.04	12.00	5.17	4.65	3.79

** $p < .01$; * $p < .01$ (2-tailed).

Supplementary Table S3

UPPS-P Impulsive Behavior Scale

Below are a number of statements that describe ways in which people act and think. For each statement, please indicate how much you agree or disagree with the statement. If you Agree Strongly circle 1, if you Agree Somewhat circle 2, if you Disagree somewhat circle 3, and if you Disagree Strongly circle 4. Be sure to indicate your agreement or disagreement for every statement below.

1 –	2 –	3 –	4 –
Agree strongly	Agree some	Disagree some	Disagree Strongly

	1	2	3	4
1. I usually think carefully before doing anything.	O	O	O	O
2. When I am really excited, I tend not to think on the consequences of my actions.	O	O	O	O
3. I sometimes like doing thinks that are a bit frightening.	O	O	O	O
4. When I am upset I often act without thinking.	O	O	O	O
5. I generally like to see things through the end.	O	O	O	O
6. My thinking is usually careful and purposeful.	O	O	O	O
7. In the hearth of an argument, I will often say things that I later regret.	O	O	O	O
8. I finish what I start.	O	O	O	O
9. I quite enjoy taking risks.	O	O	O	O
10. When overjoyed, I feel like I can’t stop myself from going overboard.	O	O	O	O
11. Once I start a project, I almost always finish it.	O	O	O	O
12. I often make matters worse because I act without thinking when I am upset.	O	O	O	O
13. I usually make up my mind through careful reasoning.	O	O	O	O
14. I generally seek new and exciting experiences and activities.	O	O	O	O
15. I tend to act without thinking when I am really excited.	O	O	O	O
16. I am a productive person who always gets the job done.	O	O	O	O
17 When I feel rejected, I will often say things that I later regret.	O	O	O	O
18. I welcome new and exciting experiences and sensations, even if they are a little frightening and unconventional.	O	O	O	O
19. Before making up my mind, I consider all the advantages and disadvantages.	O	O	O	O
20. When I am very happy, I feel like it is OK to give in to cravings or overindulge.	O	O	O	O

Factors:
Negative Urgency: 4*; 7*; 12*; 17*
Positive Urgency: 2*; 10*; 15*; 20*
Lack of Premeditation: 1; 6; 13; 19
Lack of Perseverance: 5; 8; 11; 16
Sensation Seeking: 3*; 9*; 14*; 18*
(*reversed item)

Supplementary Table S4

Affect Lability Scale – Short Form

For each statement, please rate how "generally true" each statement is. If it is uncharacteristic circle 0; if it is rather uncharacteristice cirle 1; if it is rather characteristic circle 2; and if it is very characteristic circle 3.

0 – Uncharacteristic of me	1 – rather uncharacteristice of me	2 – rather characteristic of me	3 – Characteristic of me

	0	1	2	3
1. At times I feel just as relaxed as everyone else and then within minutes I become so nervous that I feel light-headed and dizzy.	O	O	O	O
2. There are times when I have very little energy and then just afterwards I have about the same energy level as most people.	O	O	O	O
3. One minute I can be feeling OK and then the next minute Im tense, jittery, and nervous.	O	O	O	O
4. I frequently switch from being able to control my temper very well to not being able to control it very well at all.	O	O	O	O
5. Many times I feel nervous and tense and then I suddenly feel very sad and down.	O	O	O	
6. Sometimes I go from feeling extremely anxious about something to feeling very down about it.	O	O	O	O
7. I shift back and forth from feeling perfectly calm to feeling uptight and nervous.	O	O	O	O
8. There are times when I feel perfectly calm one minute and then the next minute the least little thing makes me furious.	O	O	O	O
9. Frequently, I will be feeling OK but then I suddenly get so mad that I could hit something.	O	O	O	O
10. Sometimes I can think clearly and concentrate well one minute and then the next minute I have a great deal of difficulty concentrating and thinking clearly.	O	O	O	O
11. There are times when I am so mad that I can barely stop yelling and other times shortly afterwards when I wouldnt think of yelling at all.	O	O	O	O
12. I switch back and forth between being extremely energetic and having so little energy that its a huge effort just to get where I am going.	O	O	O	O
13. There are times when I feel absolutely wonderful about myself but soon afterwards I often feel that I am just about the same as everyone else.	O	O	O	O
14. There are times when Im so mad that my heart starts pounding and/or I start shaking and then shortly afterwards	O	O	O	O

I feel quite relaxed.				
15. I shift back and forth between being very unproductive and being just as productive as everyone else.	O	O	O	O
16. Sometimes I feel extremely energetic one minute and then the next minute I might have so little energy that I can barely do a thing.	O	O	O	O
17. There are times when I have more energy than usual and more than most people and then soon afterwards I have about the same energy level as everyone else.	O	O	O	O
18. At times I feel that Im doing everything at a very slow pace but then soon afterwards I feel that Im no more slowed down than anyone else.	O	O	O	O

Subscales:
anxiety/depression (item 1, 3, 5, 6, 7)
depression/elation (item 2, 10, 12, 13, 15, 16, 17, 18)
anger (item 4, 8, 9, 11, 14).

Supplementary Table S5

Difficulties in Emotion Regulation Scale (DERS)

Please indicate how often the following statements apply to you by writing the appropriate number from the
scale below on the line beside each item.

1--------------------------2--------------------------3--------------------------4--------------------------5

almost never sometimes about half the time most of the time almost always
(0-10%) (11-35%) (36-65%) (66-90%) (91-100%)

_____ 1) I am clear about my feelings.
_____ 2) I pay attention to how I feel.
_____ 3) I experience my emotions as overwhelming and out of control.
_____ 4) I have no idea how I am feeling.
_____ 5) I have difficulty making sense out of my feelings.
_____ 6) I am attentive to my feelings.
_____ 7) I know exactly how I am feeling.
_____ 8) I care about what I am feeling.
_____ 9) I am confused about how I feel.
_____ 10) When I'm upset, I acknowledge my emotions.
_____ 11) When I'm upset, I become angry with myself for feeling that way.
_____ 12) When I'm upset, I become embarrassed for feeling that way.
_____ 13) When I'm upset, I have difficulty getting work done.
_____ 14) When I'm upset, I become out of control.
_____ 15) When I'm upset, I believe that I will remain that way for a long time.
_____ 16) When I'm upset, I believe that I will end up feeling very depressed.
_____ 17) When I'm upset, I believe that my feelings are valid and important.
_____ 18) When I'm upset, I have difficulty focusing on other things.
_____ 19) When I'm upset, I feel out of control.
_____ 20) When I'm upset, I can still get things done.
_____ 21) When I'm upset, I feel ashamed at myself for feeling that way.
_____ 22) When I'm upset, I know that I can find a way to eventually feel better.
_____ 23) When I'm upset, I feel like I am weak.
_____ 24) When I'm upset, I feel like I can remain in control of my behaviors.
_____ 25) When I'm upset, I feel guilty for feeling that way.
_____ 26) When I'm upset, I have difficulty concentrating.
_____ 27) When I'm upset, I have difficulty controlling my behaviors.
_____ 28) When I'm upset, I believe there is nothing I can do to make myself feel better.
_____ 29) When I'm upset, I become irritated at myself for feeling that way.
_____ 30) When I'm upset, I start to feel very bad about myself.
_____ 31) When I'm upset, I believe that wallowing in it is all I can do.
_____ 32) When I'm upset, I lose control over my behavior.
_____ 33) When I'm upset, I have difficulty thinking about anything else.
_____ 34) When I'm upset I take time to figure out what I'm really feeling.
_____ 35) When I'm upset, it takes me a long time to feel better.
_____ 36) When I'm upset, my emotions feel overwhelming.

Reverse-scored items (place a subtraction sign in front of them) are numbered 1, 2, 6, 7, 8, 10, 17, 20, 22, 24 and 34.

Calculate total score by adding everything up. Higher scores suggest greater problems with emotion regulation.

SUBSCALE SCORING**: The measure yields a total score (SUM) as well as scores on six sub-scales:

1. Nonacceptance of emotional responses (NONACCEPT): 11, 12, 21, 23, 25, 29
2. Difficulty engaging in Goal-directed behavior (GOALS): 13, 18, 20R, 26, 33
3. Impulse control difficulties (IMPULSE): 3, 14, 19, 24R, 27, 32
4. Lack of emotional awareness (AWARENESS): 2R, 6R, 8R, 10R, 17R, 34R
5. Limited access to emotion regulation strategies (STRATEGIES): 15, 16, 22R, 28, 30, 31, 35, 36
6. Lack of emotional clarity (CLARITY): 1R, 4, 5, 7R, 9

Total score: sum of all subscales

**"R" indicates reverse scored item

Supplementary Figure S1

Factor Structure for the Model comprising Impulsivity, Difficulties in Emotional Regulation, and Affect Lability Latent Factors

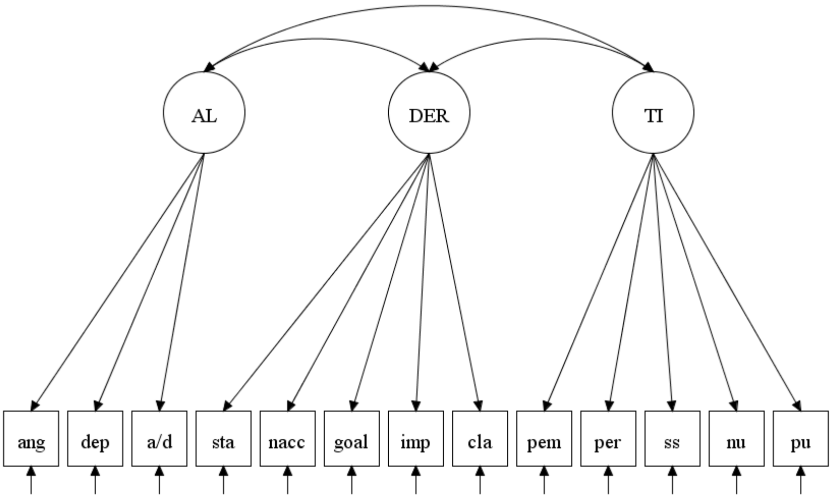

Note. ang = anger; dep = depression; a/d = anxiety/depression; sta = strategies; nacc = nonacceptace; imp = impulse; cla = clarity; pem = lack of perseverance; per = lack of premeditation; ss = sensation seeking, nu = negative urgency; pu = positive urgency; AL = affect lability; DER= dysfunctional emotional regulation; TI = trait impulsivity.

Supplementary Figure S2

Parameter Estimates for the Three-Factor Model comprising Impulsivity, Difficulties in Emotional Regulation, and Affect Lability Latent Factors

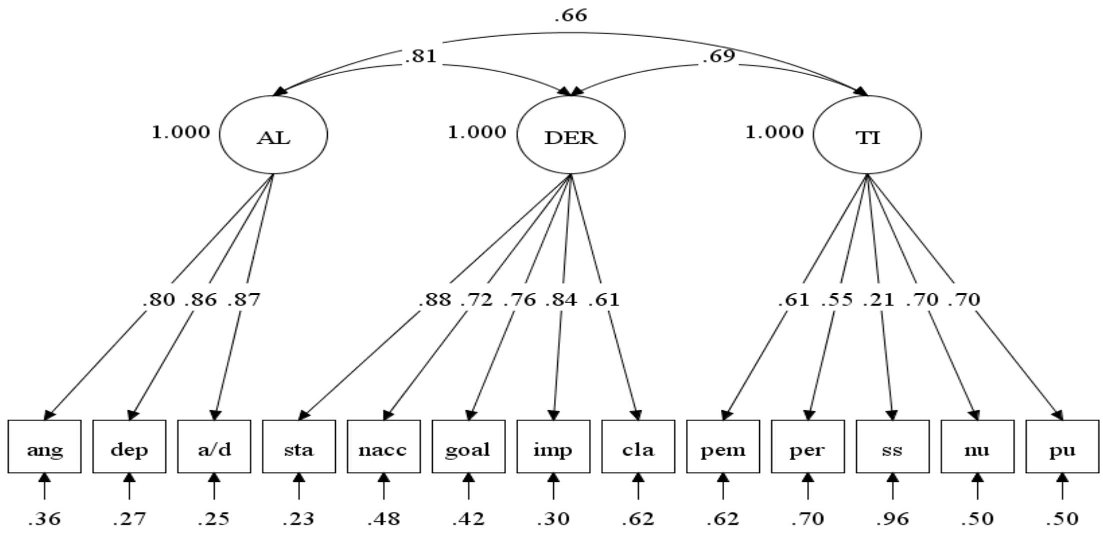

Note. ang = anger; dep = depression; a/d = anxiety/depression; sta = strategies; nacc = nonacceptace; imp = impulse; cla = clarity; pem = lack of perseverance; per = lack of premeditation; ss = sensation seeking, nu = negative urgency; pu = positive urgency; AL = affect lability; DER= dysfunctional emotional regulation; TI = trait impulsivity.
